# Health misinformation: protocol for a hybrid concept analysis and development

**DOI:** 10.12688/hrbopenres.13641.2

**Published:** 2024-01-31

**Authors:** Johanna Pope, Paula Byrne, Declan Devane, Tina D. Purnat, Maura Dowling

**Affiliations:** 1 School of Nursing and Midwifery, University of Galway, Galway, H91 TK33, Ireland; 2 Evidence Synthesis Ireland, University of Galway, Galway, H91 TK33, Ireland; 3 Health Research Board - Trials Methodology Research Network (HRB-TMRN), Galway, H91 TK33, Ireland; 4 Visiting Fellow, University of Memphis School of Public Health, Memphis, Tennessee, USA

**Keywords:** Misinformation, disinformation, health information, concept analysis

## Abstract

**Background:**

Misinformation represents a serious and growing concern for public health and healthcare health; and has attracted much interest from researchers, media, and the public over recent years. Despite increased concern about the impacts of misinformation on health and wellbeing, however, the concept of health misinformation remains underdeveloped. In particular, there is a need to clarify how certain types of health information come to be designated as “misinformation,” what characteristics are associated with this classification, and how the concept of misinformation is applied in health contexts.

**Aim:**

Developing a shared understanding of what it means for health information to be “misinformation” is an important first step to accurately identifying at-risk groups, clarifying pathways of vulnerability, and agreeing goals for intervention. It will also help to ensure that misinformation interventions are accessible, acceptable, and of benefit to the populations to which they are directed. We will therefore examine the characteristics, measurement, and applications of misinformation in health contexts.

**Methods:**

We will undertake a hybrid concept analysis, following a framework from Schwartz-Barcott & Kim (2000). This framework comprises three phases: a theoretical phase, fieldwork phase, and final analysis phase. In the theoretical phase, a search of seven electronic citation databases (PsycInfo, socINDEX, JSTOR, CINAHL, Scopus, MEDLINE and PubMed Central via PubMed, and ScienceDirect) will be conducted in order to identify original research, review, and theoretical papers, published in English between 2016 and 2022, which examine “health misinformation.” Data from the literature will be synthesised using evolutionary concept analysis methods from Rodgers (2000). In the fieldwork phase, a purposive sampling strategy will be employed to recruit stakeholders for participation in semi-structured interviews. Interviews will be analysed using thematic analysis. The final phase will integrate findings from the theoretical and fieldwork analyses.

## Introduction

Misinformation is increasingly recognised as a serious barrier to implementing public health advice and programming. For example, exposure to misinformation has been identified as contributing to reduced intention to receive vaccines ^1– 3^, engagement in unproven and potentially harmful treatments for cancer ^4^, and the decision to delay or avoid interactions with health services and institutions ^5^. This has, in turn, raised concerns that health misinformation may negatively impact the capacity of health services and public health programmes to support public well-being.

General interest in misinformation as a problem has surged over recent years: indeed the number of annual research citations containing the word “misinformation” has nearly tripled since 2016 ^6^. This likely reflects anxiety about other, related shifts in the information landscape. These shifts include, for instance, the mass shift to online information-seeking (and resultant need to adapt literacy skills to a digital environment) ^7^; the potential of the internet and social media to accelerate the spread of unreliable claims ^8^; and growing concerns about the impacts of “post-truth politics” on public decision-making ^9^.

Concerns about misinformation in health contexts have increased since the onset of the coronavirus disease 2019 (COVID-19) pandemic. In March 2020, the World Health Organization (WHO) announced an “infodemic” ^10^, resulting in significantly scaled-up investment in interventions targeting health-related misinformation ^11, 12^. Proposed interventions to combat misinformation have included, for example, “prebunking” or “psychological inoculations” ^9^, warning labels on social media posts ^13^, fact-checking programmes ^14^, and health literacy training targeting functional and critical thinking skills ^15, 16^. While these interventions have shown some promise for increasing resilience against unreliable health claims ^9, 13, 14^, much work remains to be done to understand how health misinformation spreads, who is most vulnerable to believing unreliable health claims, the best strategies for supporting individual and group resilience against health misinformation, and the best ways to measure the success of such interventions.

In addition, there remains limited consensus about what types of information constitute “misinformation” in health science. Many discussions of health misinformation have drawn on general, transdisciplinary concepts, such as the Oxford Dictionary definition (“wrong or misleading information”) ^17, 18^ or an early definition from Lewandowsky *et al*. (2012) (“any piece of information that is initially processed as valid but is subsequently retracted or corrected”) ^19, 20^. However, the relevance of these definitions for use in health science is unclear; and many studies of misinformation do not explain how they have conceptualised misinformation, instead treating it as universally understood ^20^.

Differentiating between “reliable” and “unreliable” health information in practice is complicated, not least because scientific concepts of reliability are intentionally kept flexible, and depend on evolving evidence: what is considered to be “true” about a health topic can (and should be expected to) change, both over time and in line with evolving evidence. In addition, evidence on a given topic may be uncertain, unclear, or conflicting, resulting in disagreement about which information should be regarded as “reliable.” For example, in the early months of the COVID-19 pandemic, when evidence of airborne severe acute respiratory syndrome coronavirus 2 (SARS-COV-2) was limited, many public health authorities discouraged the use of face masks outside of healthcare settings ^21^; and social media posts advising people to wear face masks in public were “debunked,” including by major authorities like the World Health Organization, as “incorrect” ^22^. However, as evidence of airborne SARS-COV-2 transmission mounted, and research emerged demonstrating that face masks could reduce the transmission of SARS-COV-2 under laboratory conditions ^23^, major public health organisations pivoted to recommending masking by the general public ^24^, despite evidentiary and expert disagreement about masks’ potential impact in real-world settings ^25– 27^. This disagreement points to theoretical tensions – as-yet unresolved – around how evidence can (or should) relate to concepts of truth in public health. Resolving these tensions is especially urgent in light of evidence that changing public health advice can have negative consequences for public trust: for example, in a qualitative study by Zhang *et al*. (2021), many participants reported perceiving the change in masking advice during the early months of the COVID-19 pandemic as an “inconsistency,” and described how this undermined their trust in public health authorities during subsequent phases of the pandemic ^28^.

Furthermore, the processes scientists use to generate and evaluate evidence are not always transparent to the public, resulting in potential uncertainty about how truth has been conceptualised or measured. The term “misinformation” (and proxy terms, such as “fake news” and “alternative facts”) have in some cases been wielded to further particular partisan agendas, or to discredit opposing viewpoints; and the term therefore remains controversial. Concerns remain about how “misinformation” items have come to be classified as such ^29^. In recent years, scholars have turned their attention to clarifying these processes and to identifying the values that inform them. For example, Vraga & Bode (2020) proposed defining misinformation as “information considered incorrect based on the best available evidence from relevant experts at the time” ^30^. As Southwell *et al*. (2022) note, this definition of misinformation has major strengths for use in science ^31^. First, it moderates the expectation that scientific information will contain any inherent quality of “correctness”: it clarifies that information can only be *regarded* as correct or incorrect, based on the available scientific evidence ^30, 31^. Secondly, it recognises that the “accuracy” of a particular piece of information may change over time ^30^; while, at the same time, affirming the value of scientific evidence in informing public decision-making ^30, 31^.

Southwell *et al*. (2022)’s subsequent definition of scientific misinformation – “publicly available information that is misleading or deceptive relative to the best scientific evidence or expertise at the time, and that counters statements by actors or institutions who adhere to scientific principles without adding accurate evidence for consideration” – draws heavily on these ideas ^31^. Their definition of misinformation is distinguished, however, by its emphasis on the value of scientific rigour (rather than simple expertise or authority) for determining the reliability of information ^31^. This is a potentially important evolution in the concept of scientific misinformation because it substantially resolves challenges around expert disagreement ^32^ and the potential of experts to themselves spread misinformation. Significantly, Southwell *et al*. (2022) also specify that, in order to be classified as misinformation, unreliable content must be publicly accessible and accessed ^31^. In this framework, misinformation is distinct from – and larger than – individual misconception or untruth ^31^. Rather, it is a “disorder of public science” (or, in other words, information which challenges our ability to achieve scientific consensus and come to “shared understandings”) ^31^.

The focus on misinformation as a threat to “shared understanding[ ]” ^31^ is echoed in other places as well; but more often it is positioned as a *symptom,* rather than a *cause,* of public dissensus. For example, over recent years, academics have described misinformation, as, variously, a symptom of breakdowns in trust between the public and social institutions, a function of increased political polarisation and decreased community cohesion, and a consequence of increased social inequality ^19, 33– 35^. It has also been hypothesised that increased social fragmentation may have catalysed a movement against scientific values, often in favour of so-called “alternative epistemologies” ^33^.

These phenomena may, of course, have serious negative consequences for implementing public health initiatives. However, some scholars have questioned the value of pathologising these “alternative epistemologies.” For example, Furman (2020a) argues that it is reasonable for an individual to reject scientific information, including health information, when the epistemic values which produced it are incompatible with their priorities ^36^. In addition, she suggests that real-world processes for evaluating the reliability of health information may, in some contexts, go beyond the “strictly epistemic”; because health-related decision-making requires people to make “all-things-considered judgements” ^36^. For this reason, information that is communicated in an accessible way ^36, 37^, transmitted by trusted sources ^37^, or identified as relevant to a person’s needs or values ^37^, may reasonably be understood to be *reliable*, even if it was not generated through scientific processes.

On the other hand, there are academics who dismiss these kinds of evaluative criteria as (for example) epistemically “lazy” ^38^, “emotive” ^39^, “irrational” ^39^, “willfully ignorant” ^40^, and “peculiar” ^37, 41^. This risks neglecting the social-ecological and cultural influences (including, for example, perspective, values, contextual relevance, and institutional trust) that impact public understandings of reliability ^19^. It may also risk creating a situation in which academics are left “talk[ing] passed” [sic] their audiences ^37^ about what it means for information to be “reliable” (or, conversely, “misinformation”). Nonetheless, much of the discourse on misinformation continues to use scientific expertise ^30^ (or its proxy, institutional affiliation ^31^) as primary indicators of content reliability. For example, recent studies ^42, 43^ and interventions ^43^ in misinformation have persisted in conceptualising “misinformation” as any item which originates from a list of pre-identified websites deemed “unreliable sources.” This disconnect should be of particular concern for those serving marginalised, underserved, and otherwise vulnerable groups, who may be more likely to have differing perspectives about, approaches to, and needs regarding the delivery of information ^18^.

One proposed solution to the challenges of determining information accuracy or reliability, outlined above, is to handle information in terms of “normativity” or “disnormativity.” In this framework, normative information represents information that is generally accepted to be true in a given context, and disnormative information is information that deviates from this accepted truth ^18, 44^. For Ruokolainen & Widén (2020), citing Haasio (2015), this approach has the advantage of allowing researchers to examine information processes without making ontological or epistemic commitments about the reliability of information ^18, 44^. It also creates conceptual space to explore how information processing, and evaluations of credibility, may be informed by different “views, norms, and values” ^44^. However, this framework may not be intuitive or well-suited to discussions about health misinformation, because health information is often *deliberately* tied to a scientific epistemology in which subjective factors are meant to be managed and minimised.

Information characteristics such as “accuracy” and “reliability” do important work in conversations about health, and there is an urgent need to develop a model for differentiating between more and less reliable health information. “Misinformation” is a useful designation for information that is not reliable. However, there is an urgent need to clarify: (1) what characteristics are accepted as aspects of health misinformation, (2) how the concept of misinformation should be operationalised for research and intervention purposes, and (3) what consequences of health misinformation have been identified. Clarifying the concept of health misinformation will be essential for developing a coherent evidence base around misinformation processes, establishing shared goals for interventions between researchers and the professionals involved in moderating health information, and ensuring that information interventions are both accessible and acceptable to the populations who will most benefit from them. Therefore, we propose to undertake a hybrid concept analysis to explore the characteristics, measurement, and applications of the concept of health misinformation.

## Protocol

### Aim

To undertake a hybrid concept analysis, to clarify the characteristics, measurement, and applications of misinformation in health contexts.

### Study design

Given the need to resolve theoretical tensions in existing academic concepts of health misinformation – and given the social nature of concepts of misinformation – we will undertake a hybrid concept analysis, following a framework from Schwartz-Barcott & Kim (2000) ^45^. Analysis will include a theoretical phase, a fieldwork phase, and a final (integrative) analysis phase ^45^.

### Theoretical phase 

***Introduction.*** In the theoretical phase, seven electronic databases ( PsycInfo, socINDEX, JSTOR, CINAHL, Scopus, MEDLINE and PubMed Central via PubMed, and ScienceDirect) will be searched for papers, including original research, review papers, and theoretical papers, published in English between 2016 and 2022, which examine health misinformation. This evidence will be synthesised using evolutionary concept analysis methods from Rodgers (2000) ^46^. Rodgers’ approach was selected because it provides a methodological foundation for analysing concepts, while allowing that the attributes of a concept may vary across social contexts, disciplines, or time ^46^. The aim of such an analysis is not to identify the essential characteristics of a concept, but, rather, to clarify the boundaries of a concept in a way that makes it relevant for current use ^46^. These features make the approach particularly well-suited for application to a concept such as misinformation, which is continuously evolving but still contains significant theoretical tensions ^46^.

***Literature search and selection.*** A three-step search strategy will be employed, following guidance on scoping searches from JBI ^47^. In the first instance, an initial search of PubMed and CINAHL will be conducted, and the results analysed to identify all relevant index terms related to the concept of misinformation ^47^. After a comprehensive list of index terms has been established, and working under guidance from an information specialist, JP will conduct a systematic search of seven electronic databases (PsycInfo, socINDEX, JSTOR, CINAHL, Scopus, MEDLINE and PubMed Central via PubMed, and ScienceDirect), including titles, abstracts, and keywords, according to a planned search strategy (available as *Extended data*) ^48^. This search will be conducted between September and November 2022. Finally, systematic searching will be augmented by a hand-search of reference lists ^47^. If the full text of a work is unavailable, JP will request a copy of the full text by contacting the work’s corresponding author.

In accordance with guidance from JBI on establishing inclusion criteria for scoping searches ^47^, the following inclusion and exclusion criteria have been established for this search.

*Eligibility criteria:*

1. Papers must be primarily focused on discussing or examining health-related misinformation. Papers which address misinformation in contexts not related to health will be excluded The following sub-criteria will be applied to determine whether a paper focused on health misinformation:
  - Misinformation will be considered to be ‘health-related’ if it pertains to information about a human mental or physical health exposure, intervention, condition, or outcome. We acknowledge that human health is complex, and influenced by a wide range of contextual factors; however, in order to facilitate focused analysis of the epistemologies that directly affect medical and public health decision-making, papers examining information about macro-level or policy phenomena that exert only indirect influences on health, or which would lie outside the scope of medical and public health practice (such as climate change, violence, or criminal justice), will be excluded. Papers which deal mainly with other types of misinformation (e.g. political) will be excluded.
  - Papers will be included only where they make specific reference to misinformation or a related term. Concept analysis is, traditionally, a semantic exercise, concerned with clarifying or developing the meaning of a specific word (in this case, ‘misinformation’); and this study is primary focused on understanding what people mean when they talk about ‘misinformation.’ However, given the relative novelty of the ‘misinformation’ label, and considering the tendency for researchers to use this term interchangeably with other, related ones, we consider it important to balance semantic considerations against the need to explore these related, yet potentially distinct, information labels. We therefore will include papers which refer to any of the following types of information by name: ‘misinformation, ‘disinformation,’ ‘fake news,’ ‘alternative facts/news,’ ‘post-truths,’ ‘conspiracy theories,’ ‘propaganda,’ ‘false information,’ ‘misleading information,’ ‘unreliable information,’ or ‘bullshit.’
  - In order to ensure richness of data, we will limit our scope to papers where health-related misinformation (or one of the related concepts listed above) was identified as a focus, theme, or sub-theme. To be eligible for inclusion under this criterion, papers must refer to misinformation (or a related construct identified above) by name in their title, abstract, or keywords, **and** dedicate substantial attention to addressing at least one of these constructs in the text body (i.e. by addressing it as a theme or sub-theme). Papers which referred to misinformation only in passing will therefore be excluded.
2. Articles must be peer-reviewed and journal-published within psychology, sociology, medicine, public health, political science, ethics, communication and media studies, or computer science. These fields were selected to support a comprehensive and multi-dimensional understanding of the academic concept of health-related misinformation, while maintaining an applied focus.
3. To be eligible for inclusion, articles must have been published between 2016 and 2022. This timeframe was chosen because of the greatly increased focus on misinformation in academic literature since the 2016 United States presidential election [8]; and the widespread consensus that public 2016 represented a pivotal turning point for public perceptions of, and consumption of, misinformation, particularly via online and social media ^49^.
4. Articles published in languages other than English will not be eligible for inclusion.

Following the search, titles and abstracts will be reviewed independently for eligibility by JP and MD, with reference to the eligibility criteria outlined above. Disagreements will be discussed and agreed, in consultation with PB. Papers will be divided into groups according to study design. In accordance with guidance from Rodgers (2000), we tentatively propose to select a sample of at least 30 papers from each design category, or roughly 20% of all papers in each design category (whichever is larger), for analysis ^46^. Sample size may be adjusted based on findings from the preliminary search. Papers will be randomly selected from each category of study design using a table of random numbers. The process of identifying and selecting works for inclusion will be documented on a PRISMA flow diagram ^50^. Screening will be managed in Rayyan software ^51^.

***Data extraction and management.*** JP will extract data, to include title of the journal, authors’ academic disciplines, year of publication, characteristics of misinformation identified by the authors, indicators used to measure whether an information item should be classified as “misinformation,” factors identified as antecedents to misinformation, identified consequences of misinformation, “surrogate terms” used by the authors to refer to misinformation, concepts identified by the authors as being related to misinformation, and any examples of health misinformation provided by the authors ^46^. This data, including verbatim quotes and page numbers ^46^, will be documented on a standardised data extraction form. A copy of this form is available as *Extended data* ^48^.

***Data synthesis.*** Following guidance from Rodgers (2000), data will be sorted into conceptual categories (including attributes, antecedents, consequences, “references” or examples, “surrogate terms,” and related terms). Subsequently, data from each category will be analysed using thematic analysis techniques from Rodgers (2000), in NVivo12 software ^46^. Attention will be paid to how the concept of health misinformation has changed over time, and to tensions in the concept of misinformation across different health-related disciplines.

### Fieldwork phase

***Study design.*** We will utilise an exploratory, qualitative design to explore public understandings of the term “misinformation.” In light of evidence suggesting that an individual’s involvement in a topic can influence their perceptions of information reliability ^52^, we hypothesise that perceptions of misinformation will also vary based on healthcare involvement. Therefore, we will conduct interviews and focus groups with members of the public, as well as interviews with professionals involved in moderating health information for public audiences. Ethical approval will be obtained from the authors’ institutional research ethics committee before commencing recruitment for fieldwork participation (ethics review is anticipated in November 2022).

***Participants.*** Eligible participants will be members of the public without training or experience in health science or research, and professionals involved in communicating health information to public audiences. To be eligible for inclusion in the study, all participants must be resident in the Republic of Ireland; however, no exclusions will be made on the basis of gender, age, level of education, health status, or location within the Republic of Ireland.

A purposive sampling strategy will be employed to recruit participants. Members of the public will be recruited via posts on social media. In addition, we will identify gatekeepers within historically marginalised communities, including through community groups serving minority groups, and request assistance from these gatekeepers with circulating study information. Health communicators will be recruited to participate via professional organisations (e.g. National Association of General Practitioners, Irish Medical Council). Participant information sheets will be sent to relevant bodies and, where agreed, circulated amongst members, along with an invitation to contact the primary investigator for further information about the study. Snowball sampling will be employed to supplement this participation, as needed.

**Data collection and management**

Before enrolling in the study, participants will receive a participant information sheet containing information about the aims of the study, and a description of what participation will entail. Participants will also be apprised of the voluntary and anonymous nature of the research, their right to withdraw in part or in full at any point during participation or up to one week thereafter, and all relevant data storage and usage policies. Prior to engaging in interviews, the researcher will obtain written consent using a consent form. Consent forms will be sent to individuals expressing interest in the study via e-mail,, and returned signed, before each interview.

Due to the national focus of this work, and ongoing precautions related to the COVID-19 pandemic, interviews will be conducted via phone or Zoom, per participants’ preferences, between September and November 2022. Focus groups will be conducted in person, as possible, or over Zoom; the exact location and timing of these focus groups will be determined by pandemic-related considerations, as well as by participant availability and preference.

Interviews will begin with a brief reiteration of the research aims and what participation will entail. Participants will again be verbally reminded of their right to withdraw from the interview, in part or in full, at any time. After receiving this information, participants will be asked to provide verbal consent to proceed to the interview. This verbal consent will be recorded using a Dictaphone, and held in a separate recording from the main interview.

Interviews will also be audio-recorded using a Dictaphone. These recordings will subsequently be transcribed and pseudo-anonymised by JP. The interviewer (JP) will be guided by a semi-structured topic guide developed by an interdisciplinary team, and will include questions about participants’ attitudes towards the concept of misinformation and perceptions of information reliability. Participants will also be asked about their information-seeking behaviours, concepts of source and content reliability, and experiences with health-related misinformation. Full copies of the interview topic guides are available on OSF ^48^.

Data collection will continue iteratively until it is agreed that data adequacy, or sufficiently rich data, has been collected ^53^. It is estimated that the study will include approximately 45 participants, including roughly 25 members of the lay public and 20 health professionals. Iterative data collection will continue until data adequacy is achieved ^53^.

***Data analysis.*** The analysis will proceed from a constructivist perspective, assuming that concepts of misinformation are socially constructed and may vary across contexts ^46^. Given this perspective, the inductive nature of the larger analysis ^45, 46^, and the need for an analytical method for identifying patterns across disciplines without making judgements about their ontological value, Braun & Clarke’s (2006)’s thematic analysis framework has been identified as the most appropriate method for analysis ^54, 55^.

### Final analytical phase

This phase will consist of further interrogation and integration of the findings from prior phases, to clarify the characteristics, measurement, and applications of the concept of health misinformation.

During this phase, we will first focus on articulating a clear definition of health misinformation. In line with guidance from Schwartz-Barcott & Kim (2000), attention will also be paid at this stage to how definitions of health misinformation have evolved over time and across disciplines, as well as how theoretical understandings of health misinformation may overlap with – or differ from – the concepts observed in fieldwork ^45^. We will also outline how health misinformation has been operationalised for use in research to date, and provide recommendations for refining the tools and indicators for identifying health misinformation in light of our theoretical and fieldwork findings ^45^. Finally, we will discuss the applicability and relevance of the concept of misinformation in health contexts, and provide some direction for future inquiry and refinement ^45^.

### Dissemination 

A report of the findings will be prepared for open access journal publication. We will also disseminate findings through infographic summaries, online media, and broadcast media.

## Discussion

### Strengths and limitations

This research aims to clarify the characteristics, measurement, and applications of the concept of health misinformation. Therefore, the literature phase of this study has focused on discussions of health-related misinformation, excluding other types of misinformation. This facilitates targeted examination of how the concept of misinformation has been conceptualised and applied in the context of health, healthcare, and health policy – and allows us to focus on theoretical models that are most relevant to health science epistemologies. However, given that concepts of health misinformation are likely influenced by discourses on misinformation being generated in other disciplines, this approach may also obscure some applications of the concept.

On the other hand, Schwartz-Barcott & Kim (2000)’s hybrid concept analysis framework encourages multidimensional (and multi-contextual) exploration of a concept ^45^. This approach may be particularly valuable for this analysis, given the growing consensus that perceptions of misinformation can be influenced by a wide range of contextual factors ^18, 19, 33^. The approach also allows for significant exploration of lay concepts of misinformation, which may be expected to differ – perhaps even significantly – from those presented in academic texts.

### Study status

An initial search of MEDLINE and PubMed Central (via PubMed) and CINAHL was conducted in September 2022. A complete systematic search of targeted databases is underway, as of October 2022.

## Conclusion

Exposure to unreliable health claims carries risks for individual health and health-related decision-making; and may have further, indirect consequences for public health programmes, policies, and health systems. “Misinformation” is a potentially useful concept for communicating about the reliability of health claims. However, there remains a lack of clarity – and, in some cases, active disagreement – about how misinformation should be identified, characterised, or addressed. Clarifying the concept of misinformation will allow for more accurate identification of at-risk groups, pathways of vulnerability, and potentially useful goals for intervention. It will also facilitate trust between health authorities and their audiences; and help to ensure that misinformation interventions reflect the information needs, values, and goals of the populations to which they are directed.

## Data Availability

No underlying data is associated with this protocol. Open Science Framework: Health Misinformation: Protocol for a Hybrid Concept Analysis and Development. https://doi.org/10.17605/OSF.IO/6UDVE ^48^. This project contains the following extended data: Supplementary File 1_ Literature Search Strategy.docx Supplementary File 2_ Standard Data Extraction Form.docx Supplementary File 3_ Topic Guides.docx Data are available under the terms of the Creative Commons Zero "No rights reserved" data waiver (CC0 1.0 Public domain dedication).
