# Supplementary material for: Health misinformation: protocol for a hybrid concept analysis and development
Source: HRB Open Res. 2024 Jan 31;5:70. Originally published 2022 Nov 1. [Version 2] doi: 10.12688/hrbopenres.13641.2 (PMC11621614; doi:10.12688/hrbopenres.13641.2)
Supplement: Peer review history

# Reviewer report: Reviewer response for version 2

**DOI:** 10.21956/hrbopenres.15185.r42487

**Published:** 2024-12-05

**Authors:** Sanjaikanth E Vadakkethil Somanathan Pillai

**Affiliations:** 1 University of North Dakota, Grand Forks, North Dakota, USA

Strengths:

 1. The focus on health misinformation is highly relevant and timely, especially in the post-COVID-19 era, where public health misinformation has become widespread.

 2. The use of Schwartz-Barcott & Kim’s hybrid concept analysis framework enables a comprehensive, multidimensional exploration of the concept.

 3. The inclusion of multiple databases (e.g., PsycInfo, MEDLINE, Scopus) ensures an extensive and diverse literature search.

 4. Efforts to include marginalized communities in the fieldwork enhance the study’s relevance and applicability, contributing to its depth and inclusivity.

 Weaknesses:

 1. The protocol lacks clarity on whether the focus is specifically on "public health misinformation" or "health misinformation" more generally. This inconsistency could lead to confusion regarding the scope and focus of the research.

 2. Recruitment through professional organizations and social media may introduce selection bias, potentially affecting the representativeness of the sample.

 3. The protocol does not clearly define how "misinformation" will be operationalized or measured. Without clear definitions, the study may face issues with ambiguous or inconsistent findings.

 4. There is limited exploration of how misinformation differs from disinformation and other related terms. This distinction is important, as each term carries different implications.

 5. The exclusive focus on participants from the Republic of Ireland may limit the applicability of the findings to other regions with different dynamics regarding health misinformation.

 6. The distinction between PubMed as a search engine and the databases it queries (e.g., MEDLINE, PubMed Central) is not clearly explained, which could cause confusion.

 Overall Suggestion: The strengths of the study outweigh its weaknesses. Therefore, I recommend proceeding with the publication of this research.

Is the study design appropriate for the research question?

Yes

Is the rationale for, and objectives of, the study clearly described?

Yes

Are sufficient details of the methods provided to allow replication by others?

Yes

Are the datasets clearly presented in a useable and accessible format?

Yes

Reviewer Expertise:

Misnformation detection, COvid-19 Fake News, AI/ML, Mobile Network comunication.

I confirm that I have read this submission and believe that I have an appropriate level of expertise to confirm that it is of an acceptable scientific standard.

# Reviewer report: Reviewer response for version 1

**DOI:** 10.21956/hrbopenres.14915.r34328

**Published:** 2023-08-25

**Authors:** Mark Petticrew

**Affiliations:** 1 Department of Public Health, Environments and Society, London School of Hygiene and Tropical Medicine (LSHTM), London, UK

This is a useful review aiming to assess - via review and primary research - how the concept of misinformation is used in public health.

 My main comment is that the health focus of the project is not always consistent - what aspect of health is the focus? I think the protocol could clarify how the researchers are defining public health – what topics in this very broad field are relevant for inclusion?  In other places, "public" health does not seem to be the focus: in the protocol methods section, it mentions ‘health misinformation’ which is different from (and much broader than) ‘public health misinformation’. Again, elsewhere there is mention of ‘public health interventions’  - which is a much narrower and very different focus. So it is not always clear what the focus of the review and the qualitative research might be.

 The inclusion criteria could similarly be clarified. At the minute they contain the criterion: “Articles must examine or apply a concept of health-related misinformation. Studies examining mis-information in contexts not related to health will be excluded.” It is a bit unclear on this basis whether the review will include ‘articles’ (which could mean any paper describing or analysing or commenting on misinformation) – or just "studies"  - which analyse misinformation. If it is ‘studies’ rather than ‘articles’ that are to be included, then the types of study which are eligible for inclusion should be described (whether qualitative or quantitative,  or whatever, and of what design). Without this the review might risk including any type of commentary, or any article which mentions misinformation in passing, but which includes no in-depth exploration or anaysis of the concept.

Is the study design appropriate for the research question?

Yes

Is the rationale for, and objectives of, the study clearly described?

Yes

Are sufficient details of the methods provided to allow replication by others?

No

Are the datasets clearly presented in a useable and accessible format?

Not applicable

Reviewer Expertise:

Public health

I confirm that I have read this submission and believe that I have an appropriate level of expertise to confirm that it is of an acceptable scientific standard.

# Reviewer report: Reviewer response for version 1

**DOI:** 10.21956/hrbopenres.14915.r35148

**Published:** 2023-08-11

**Authors:** Ivan Herrera-Peco, Beatriz Jiménez-Gómez, Invención Fernández-Quijano

**Affiliations:** 1 Nursing Department, Faculty of Medicine, Alfonso X El Sabio University, Madrid, Spain; 2 Department of Nursing, Human Nutrition and Dietetics, Universidad Europea de Madrid SLU, Madrid, Community of Madrid, Spain; 3 Psychology. Faculty of Medicine, Universidad Alfonso X el Sabio, Villanueva de la Cañada, Community of Madrid, Spain

After reviewing the protocol, the following points require clarification:

**1.- Literature search and selection**

 In your first phase, you indicate that PubMed is a database. Can you explain why you reach this conclusion if PubMed is the search engine for the MedLine and PubMed Central databases?

 Please, review this information to clarify it for future readers.

**2. Field Study:**

**2.1. Study design**

 You indicate that interviews will be conducted by telephone or Zoom, due to the precautions to be maintained by the COVID19 , during the months of September-November 2022. At the present time, August 2023, I understand that you have already conducted these interviews. Or is it a mistake to have indicated the date in your manuscript?

**2.2. Data collection and management**

 It would be necessary to indicate how the authors will obtain consent for the participation of the subjects.

 If the interview is by Zoom, will they record the interview?

 If it is by telephone, how do you plan to do it?

 Will the documentation be sent to them by e-mail for them to return it signed?

**3. Discussion**

 When assessing the limitations of the study, elements such as, among others, the study design itself, the sampling technique or the sample selected should be considered.

Is the study design appropriate for the research question?

Partly

Is the rationale for, and objectives of, the study clearly described?

Yes

Are sufficient details of the methods provided to allow replication by others?

Partly

Are the datasets clearly presented in a useable and accessible format?

Not applicable

Reviewer Expertise:

Health misinformation, and disinformation, on Social media.

We confirm that we have read this submission and believe that we have an appropriate level of expertise to confirm that it is of an acceptable scientific standard, however we have significant reservations, as outlined above.
